# Erratum: Genetic modulation of training and transfer in older adults: BDNF Val^66^Met polymorphism is associated with wider useful field of view

**DOI:** 10.3389/fpsyg.2013.00720

**Published:** 2013-10-08

**Authors:** Lorenza S. Colzato

**Affiliations:** Cognitive Psychology Unit and Leiden Institute for Brain and Cognition, Leiden UniversityLeiden, Netherlands

**Keywords:** videogame, brain-derived neurotrophic factor, useful field of view, brain training, aging

A commentary on Genetic modulation of training and transfer in older adults: BDNF Val^66^Met polymorphism is associated with wider useful field of view by Colzato, L. S., van Muijden, J., Band, G. P. H., and Hommel, B. (2011) Front. Psychol. 2:199. doi: 10.3389/fpsyg.2011.00199

Please find below a list of corrections made to this article.

On page 4, right column, under Results, second sentence, after “more important for present purposes” the text “assessment was involved in a three-way interaction with group and BDNF, *F*_(1, 56)_ = 3.77, *p* = 0.049, *MSE* = 6385.66, η^2^_*p*_ = 0.058.” should read “assessment was involved in a tended to be significant three-way interaction with group and BDNF, *F*_(1, 56)_ = 3.77, *p* = 0.057, *MSE* = 6385.66, η^2^_*p*_ = 0.058”

On page 4, Table 1, third column, seventh row, the text “114 (26)” should read “111 (28)”.

On page 4, Table 1, third column, eight row, the text “−30” should read “−33.”

